# Comparative Analysis of Lactobacillus Starter Cultures in Fermented Camel Milk: Effects on Viability, Antioxidant Properties, and Sensory Characteristics

**DOI:** 10.3390/foods13223711

**Published:** 2024-11-20

**Authors:** Amal Bakr Shori

**Affiliations:** Department of Biological Sciences, Faculty of Science, King Abdulaziz University, Jeddah 21589, Saudi Arabia; shori_7506@hotmail.com

**Keywords:** *L. rhamnosus*, *L. casei*, *L. plantarum*, fermentation, camel milk, antioxidants, storage

## Abstract

This research evaluated the impact of *Lactobacillus* starter cultures on post-acidification, viable cell counts (VCC), antioxidant activities (such as DPPH radical scavenging, Ferric Reducing Antioxidant Power (FRAP), and Ferrous Ion Chelating (FIC) activity), and sensory attributes of fermented camel milk (FCM) over a 21-day period at 4 °C. FCM was prepared with *Streptococcus thermophilus* and *Lactobacillus delbrueckii* subsp. *lactis* (control), as well as with three different *Lactobacillus* starter cultures individually: *Lacticaseibacillus rhamnosus*, *Lacticaseibacillus casei*, and *Lactiplantibacillus plantarum*, in co-cultures with *S. thermophilus* and *L. delbrueckii* subsp. *lactis*. The findings indicated that FCM with *L. rhamnosus* experienced the most significant pH decrease (*p* < 0.05) throughout the storage period. *L. plantarum*-FCM maintained the highest VCC (*p* < 0.05) compared with the other samples. Additionally, all three *Lactobacillus* strains showed significantly higher (*p* < 0.05) DPPH radical scavenging and FRAP compared with the control by the end of the storage. However, *L. casei* exhibited the greatest FIC activity. Among the samples, *L. plantarum* was rated highest in taste, flavor, and overall preference. In conclusion, the incorporation of these *Lactobacillus* strains into camel milk during fermentation improved bacterial viability, enhanced antioxidant properties, and boosted sensory qualities, especially for flavor and texture, positioning it as a promising functional food product.

## 1. Introduction

Antioxidants play a vital role in reducing the likelihood of chronic illnesses, such as cancer and heart disease, caused by free radical reactions [1,2,3]. Dairy products are a valuable source of antioxidants, which contribute to their potential health benefits. These products are rich in different antioxidant substances, including milk caseins and whey proteins, which play a role in neutralizing harmful free radicals [4]. Additionally, dairy products are rich in essential antioxidants such as vitamins A and E, as well as carotenoids like beta-carotene [5]. Milk also provides low-molecular-weight thiols, ascorbic acid (vitamin C), and several enzyme systems, including superoxide dismutase and catalase, which further enhance their antioxidant capacity [6]. These naturally occurring antioxidants in dairy products help protect against oxidative stress and support overall health, making them an important component of a balanced diet.

Camel milk typically appears as an opaque white liquid with a flavor profile that is both salty and sweet. While its overall composition is like that of cow’s milk, there are notable differences within the molecular composition of its proteins, fats, and minerals [7]. It has a good level of protein (3.1%), α-lactalbumin (27%), serum albumin (26%), immunoglobulins (18%), lactose (4.4%), fat (3.5%), and ash (0.79%) [8]. In addition, camel milk fat is abundant in polyunsaturated fatty acids [9]. It is abundant in vital nutrients and minerals, such as manganese, copper, iron, sodium, potassium, and zinc [10]. Additionally, it possesses antioxidant properties that contribute to a reduction in free radicals and reactive oxygen molecules [6]. This potent antioxidant effect may be attributed to its high vitamin C concentration, which is threefold greater than that found in cow’s milk [6].

Probiotics are described as “a live microorganism present in dietary supplements which confers a health benefit to the host when administered in adequate amounts” [11]. Milk is an appropriate medium for inoculating with probiotic cultures, since it is widely consumed by a substantial number of people worldwide due to its considerable nutritional benefits [12]. It also has bioactive peptides that are produced by the probiotic cultures during proteolytic activity, which may improve cardiovascular health, bones, immunity, digestion, and bowel health [13]. In addition, these bioactive peptides were found to possess antioxidant activity, making fermented milk more popular as a functional food [14]. Numerous studies have investigated how modifying starter cultures, such as *Lactobacillus rhamnosus*, *L. casei*, and *L. plantarum* [15,16], can enhance the nutritional and therapeutic properties of fermented milk. The purpose of this research was to assess the impact of these probiotic strains, specifically *L. rhamnosus*, *L. casei*, and *L. plantarum*, when used in combination with *Streptococcus thermophilus* and *L. delbrueckii* subsp. *lactis*. The study focused on assessing how these strains affected post-acidification, the survival rate of lactic acid bacteria, and the antioxidant properties in fermented camel milk (FCM) throughout a storage period of 0, 7, 14, and 21 days. Additionally, a sensory assessment of all FCM samples was performed on the initial day of storage.

## 2. Materials and Methods

### 2.1. Materials and Chemicals

Isolated strains of *Streptococcus thermophilus* St1342, *Lactobacillus delbrueckii* subsp. *lactis* ATCC 7830, *Lacticaseibacillus casei* ATCC 393, *Lactiplantibacillus plantarum* ATCC 14917, and *Lacticaseibacillus rhamnosus ATCC 53103* were sourced from the Microbiology National Committee at Ain Shams University. Fresh and pasteurized camel milk (Al-turath^®^) was sourced from a nearby supermarket in Jeddah, Saudi Arabia, containing 11.91% total solids, 4.33% fat, and 3.35% protein. All chemicals employed in the study were bought from Sigma-Aldrich Co., Saint Louis, MO, USA.

### 2.2. Preparation of Starter Culture

Bacterial strains were preserved at −80 °C and reactivated through subculturing in MRS broth. Incubation was conducted at 37 °C for *S. thermophilus*, *L. casei*, *L. plantarum*, and *L. rhamnosus*, while *L. delbrueckii* subsp. *lactis* was incubated at 42 °C. To prepare starter cultures, 1% (*v*/*v*) of each strain was inoculated into 10 mL of reconstituted skim milk (RSM) enriched with 2% glucose and 1% yeast extract [15].

### 2.3. Preparation of Fermented Camel Milk

Four types of fermented camel milk (FCM) were prepared for this study: one with *L. rhamnosus*, another with *L. casei*, and a third with *L. plantarum*, each combined with *S. thermophilus* and *L. delbrueckii* subsp. *lactis*. A control FCM was also made, containing only *S. thermophilus* and *L. delbrueckii* subsp. *lactis*. The preparation followed the protocol outlined by Shori et al. [16]. Each FCM sample was made by blending 98 mL of pasteurized full-cream milk with a 2% starter culture. The starter culture consisted of individual strains at a concentration of 10^5^ CFU/mL. For the control FCM, the starter cultures were mixed in equal ratios, whereas for the Lactobacillus FCM, the mix was at a 1:1:1 ratio. The mixture was incubated at 42 °C for 3 h to allow fermentation, which was then terminated by cooling the samples in a cold-water bath at 4 °C for 1 h. All FCM samples were subsequently stored at 4 °C for 21 days.

### 2.4. Determination of pH and Titratable Acid (TA)

The pH and titratable acidity (TA) of the FCM samples were assessed using the procedure outlined by Muniandy et al. [17].

### 2.5. Viable Cell Counts (VCC) of LAB in FCM

A 1 mL aliquot from each FCM sample was combined with 9 mL of sterile buffered peptone water (0.15% concentration). Serial dilutions were then performed up to a 10^5^-fold dilution using the same buffered peptone water, following the approach detailed by Muniandy et al. [17]. A 1 mL sample of diluted yogurt was cultured on M17 agar to isolate *S. thermophilus* and on MRS agar for *Lactobacillus* spp. [18]. The plates were incubated at 37 ± 1 °C for 48 h. The viable cell count (VCC) was determined using the formula:

CFU/mL = (Number of colonies) × (dilution factor)/(volume of sample in mL)

### 2.6. Preparation of FCM Water Extracts

For each FCM sample, 10 g was combined with 2.5 mL of distilled water, as outlined by Muniandy et al. [19]. The solution was then adjusted to a pH of 4.0 using 0.1 M hydrochloric acid (HCl) and placed in a water bath at 45 °C for 10 min. Following this, the mixture was centrifuged at 5000× *g* and 4 °C for 10 min. The resulting supernatant was neutralized to pH 7.0 with 0.1 M sodium hydroxide (NaOH) and subjected to a second round of centrifugation under the same conditions. The clear supernatant obtained from this final centrifugation was collected for subsequent analysis.

### 2.7. Antioxidant Properties

#### 2.7.1. DPPH Radical Scavenging Assay

A 3 mL solution of DPPH reagent (60 mM), prepared in 95% ethanol, was mixed with 250 μL of FCM water extract [19]. The resulting mixture was left to incubate at room temperature for 1 h in the dark. The absorbance at 517 nm was then recorded using a Genesys 10UV spectrophotometer (Thermo Fisher Scientific, Waltham, MA, USA), with a control sample containing 250 μL of ethanol in place of the extract. The percentage of radical scavenging activity was determined using the following formula:

Radical scavenging activity (%) = [(Absorbance of control at 517 nm − Absorbance  of sample at 517 nm)/Absorbance of control at 517 nm] × 100

#### 2.7.2. Ferrous Ion Chelating (FIC) Ability Assay

For the assay, a 2 mM solution of iron (II) sulfate hydrate (FeSO_4_.xH_2_O_2_) and a 5 mM ferrozine solution were each diluted 20-fold before use [15]. A 1 mL of the diluted FeSO_4_.xH_2_O solution was combined with 1 mL of FCM water extract and 1 mL of the diluted ferrozine solution. This mixture was then incubated for 10 min at 25 °C. The absorbance at 562 nm was recorded using a Genesys 10UV spectrophotometer, with a control measurement taken using 1 mL of distilled water in place of the extract. The free iron chelation (FIC) ability of the samples was determined using the following formula:

FIC ability (%) = [(Absorbance of control at 562 nm − Absorbance of sample at  562 nm)/Absorbance of control at 562 nm] × 100

#### 2.7.3. Ferric Reducing Antioxidant Potential (FRAP) Assay

A FRAP reagent was prepared by combining 3.6 mL of a solution containing 300 mM acetate buffer, 8 mM 2,4,6-tri(2-pyridyl)-s-triazine (TPTZ), and 20 mM FeCl_3_ in a 10:1:1 ratio. This reagent was then mixed with 400 μL of either FCM water extract or a standard iron (II) sulfate heptahydrate (FeSO_4_.7H_2_O) solution, with concentrations ranging from 0.3 to 1.0 μg/mL, as described by Muniandy et al. [19]. The resulting mixture was incubated at 37 °C for 10 min in a water bath. After incubation, the absorbance was measured at 593 nm using a Genesys 10UV spectrophotometer. The results were quantified by comparing with a standard curve of FeSO_4_ × 7H_2_O and expressed as millimolar Fe^2+^ equivalents per milliliter (mM Fe^2+^ E/mL).

### 2.8. Sensory Properties

On the first day of storage at 4 °C [16], a sensory evaluation of the FCM was conducted by a panel of 13 untrained individuals, comprising both students and faculty members from the Department of Biological Sciences at King Abdulaziz University [15]. The participants, aged between 20 and 45 years (average age 22), rated the FCM on six sensory attributes: taste, color, flavor, aroma, texture, and overall preference. Each attribute was assessed using a 10-hybrid hedonic scale [20], where 10 represented excellent quality, 9 indicated high acceptability, 8 denoted acceptable, 7 was moderately acceptable, 6 slightly acceptable, 5 slightly unacceptable, 4 moderately unacceptable, 3 unacceptable, 2 highly unacceptable, and 1 indicated rejection.

### 2.9. Statistical Analysis

Three experimental batches were prepared in duplicate (n = 3 × 2). Results are expressed as mean ± standard error of the mean (SEM). To assess the significance of differences between the means, one-way analysis of variance (ANOVA) was employed, with a significance threshold set at *p* < 0.05. Statistical analyses were carried out using IBM SPSS Statistics version 20.0 (IBM Corp; Armonk, NY, USA).

## 3. Results and Discussions

### 3.1. Post-Acidification Activity in FCM

**Figure 1:**
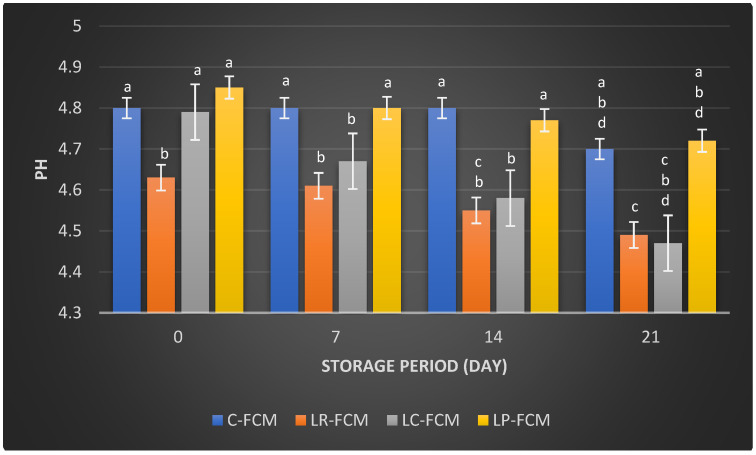
The pH values of fermented camel milk (FCM) with the addition of *L. rhamnosus* (Lr), *L. casei* (Lc), or *L. plantarum* (Lp), compared with control (C) FCM over 21 days of refrigerated storage at 4°C. Data are presented as mean ± SEM. abcd means with different superscript letters indicate the level of significance at *p* < 0.05 compared with control at the same storage period.

**Figure 2:**
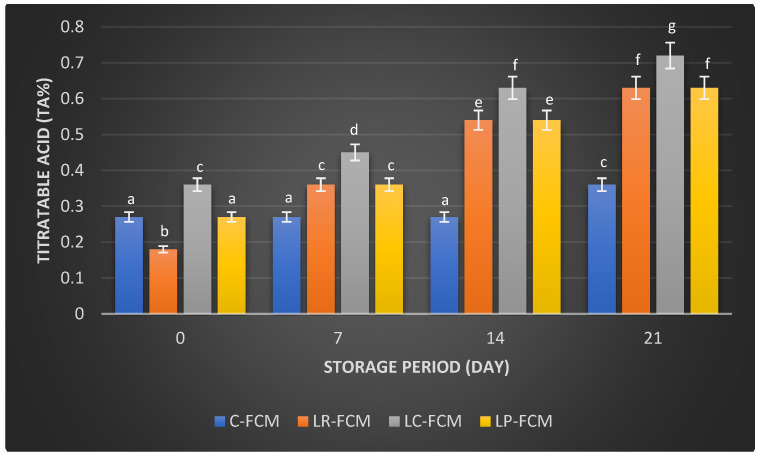
Titratable acidity (TA%) of fermented camel milk (FCM) with the addition of *L. rhamnosus* (Lr), *L. casei* (Lc), or *L. plantarum* (Lp), compared with control (C) FCM over 21 days of refrigerated storage at 4 °C. Data are presented as mean ± SEM. abcdefg means with different superscript letters indicate the level of significance at *p* < 0.05 compared with control at the same storage period.

Figure 1 and Figure 2 present the pH values and titratable acid (TA%), respectively, of FCM with different starter cultures in comparison with control over 21 days of cold storage at 4 °C. The control FCM maintained a relatively stable pH of 4.8 ± 0.005, slightly decreased (*p* < 0.05) to 4.7 ± 0.045 by day 21 of storage (Figure 1). In contrast, FCM samples with different starter cultures showed a gradual decline (*p* < 0.05) in pH throughout the storage period, which in the presence of *L. rhamnosus* dropped from 4.63 to 4.49, *L. casei* from 4.79 to 4.47, and *L. plantarum* from 4.85 to 4.72. These results indicate that *L. rhamnosus* and *L. casei* demonstrate more significant (*p* < 0.05) pH reductions compared with *L. plantarum* (Figure 1).

The titratable acid (TA) values of samples showed distinct patterns (Figure 2). The control FCM had a stable TA of 0.27% until day 14, which increased (*p* < 0.05) to 0.36% by day 21. In contrast, TA in FCM with *L. rhamnosus* and *L. plantarum* started at 0.18% and 0.27%, respectively, and rose sharply to 0.63% by day 21 (*p* < 0.05). Similarly, the TA of FCM with *L. casei* followed a similar trend, which increased (*p* < 0.05) from 0.36% to 0.72% over the same duration (Figure 2).

Post-acidification is the quantity of acid that accumulates during storage. It can significantly impact the product’s quality by enhancing the fermented milk flavor and increasing its health benefits through the production of beneficial metabolites [21]. During refrigeration, fermented milk can experience post-acidification due to the continued metabolic activity of LAB [12]. Even at low temperatures (0–5 °C), the enzyme β-galactosidase remains active, allowing bacteria to further break down lactose [14]. This process results in the production of various acids, including lactic, acetic, citric, butyric, as well as compounds like acetaldehyde and formic acid, which are generated as metabolic byproducts of the LAB [14]. The pH and titratable acid (TA) values of FCM samples over 21 days at 4 °C revealed significant changes due to the fermentation process and post-acidification facilitated by different starter cultures. This study showed that the presence of *L. rhamnosus* or *L. casei* accelerated the reduction in pH in FCM during storage. This could be due to the stimulation of these bacteria’s growth and their metabolism [12]. FCM with *L. plantarum* exhibited no significant differences (*p* > 0.05) in pH changes compared with the control during storage; however, TA increased significantly (*p* < 0.05) over three weeks of refrigeration (Figure 1 and Figure 2). This increase in TA can be attributed to the high proteolytic activity of *L. plantarum* [22], which breaks down proteins into peptides and amino acids. The production of these amino acids may contribute to a slight alkalinization of the growth medium [14]. Consequently, the measured pH of the yogurt during storage reflects the net effect of both acid production and residual amino groups [14]. It is also important to note that titratable acidity quantifies total acidity, incorporating various acids, such as organic acids and amino acids, without distinguishing between them [21]. The minimal changes in pH and TA in control FCM (Figure 1 and Figure 2) indicated low viability of *S. thermophilus* and *L. delbrueckii* subsp. *Lactis*, as well as the limited metabolic activity of these strains. This is aligned with previous studies on fermented camel milk, which reported minimal pH changes during storage when *S. thermophilus* and *L. delbrueckii* ssp. *bulgaricus* were present [20]. El-Sayed et al. [23] and Shori et al. [15] observed a significant pH decrease and TA increase in FCM inoculated with Lactobacillus strains, affirming these cultures’ effective acid production and fermentation capabilities. In addition, the higher TA in *L. casei*-FCM (Figure 2) was consistent with a previous study that demonstrated significant improvements in TA in camel milk fermented with *L. casei subsp. casei B*-1922 [23]. Compared with our previous study on yogurt made from cow milk using similar *Lactobacillus* starter cultures, cow milk yogurt exhibited a higher titratable acidity than fermented camel milk [15]. This difference may be attributed to the higher buffering capacity of camel milk, which could better resist changes in pH during post-acidification [24].

### 3.2. Survival Rate of Lactic Acid Bacteria in FCM

Table 1 illustrates the viable cell counts of *S. thermophilus* and *Lactobacillus* spp. in FCM with different starter cultures (*L. rhamnosus*, *L. casei*, and *L. plantarum*) compared with control FCM over 21 days of cold storage at 4 °C. The control FCM showed a significant decline in *S. thermophilus* counts, starting at 8.15 ± 6.42 log CFU/mL on day 1 and plummeting (*p* < 0.05) to 6.00 ± 0.57 log CFU/mL by day 21. In contrast, FCM with *L. rhamnosus* maintained a high survival rate of *S. thermophilus*, which peaked (*p* < 0.05) at 8.82 ± 1.77 log CFU/mL on day 7 before decreasing to 8.54 ± 3.32 log CFU/mL (*p* < 0.05) by day 21 (Table 1). *S. thermophilus* in FCM with *L. casei* started at 8.64 ± 1.91 log CFU/mL, peaked at 8.68 ± 4.74 log CFU/mL (*p* > 0.05) on day 7, and then declined to 8.15 ± 0.28 log CFU/mL (*p* < 0.05) by day 21. The sample with *L. plantarum* showed an initial *S. thermophilus* count of 8.43 ± 1.13 log CFU/mL, increased (*p* < 0.05) to 8.73 ± 4.81 log CFU/mL on day 7, and then dropped (*p* < 0.05) to 8.29 ± 0.35 log CFU/mL by day 21 (Table 1).

For the survival rate of *Lactobacillus* spp., the control FCM exhibited relatively stable counts, which started at 6.00± 0.57 log CFU/mL on day 1 and peaked (*p* < 0.05) at 6.23 ± 4.16 log CFU/mL by day 7, and then slightly decreased (*p* < 0.05) to 6.04± 1.52 log CFU/mL by day 21 (Table 1). For FCM with *L. rhamnosus*, the initial *Lactobacillus* spp. count of 6.35± 0.21 log CFU/mL rose significantly (*p* < 0.05) to 6.67 ± 4.17 log CFU/mL on day 7 but declined (*p* < 0.05) to 6.13 ± 0.35 log CFU/mL by day 21. The sample with *L. casei* showed an initial high *Lactobacillus* spp. count of 6.87 ± 3.25 log CFU/mL, which decreased (*p* > 0.05) to 6.41 ± 1.34 log CFU/mL by day 7 and further to 6.28 ± 0.14 log CFU/mL (*p* > 0.05) by day 21 (Table 1). Among the samples, FCM with *L. plantarum* showed the highest initial survival rate of *Lactobacillus* spp., which started at 6.94 ± 0.49 log CFU/mL on day 1 and maintained counts of 6.7 ± 1.20, 6.63 ± 2.47 (*p* < 0.05), and 6.5 ± 3.04 log CFU/mL by days 7, 14, and 21, respectively (Table 1).

The present results are aligned with previous studies that demonstrated the viability of Lactobacillus strains in fermented dairy foods [15,23,25]. For instance, studies have reported that *L. rhamnosus*, *L. casei*, or *L. plantarum* exhibited robust growth and survival rates in fermented milk [15,23,26,27], similar to our findings, where all *Lactobacillus* spp. maintained high cell counts with substantial counts up to day 21 (Table 1). Soleymanzadeh et al. [25] found that the *L. plantarum* SM06 count in fermented camel milk was the highest (9 log CFU/mL) compared with *L. kefiri*, *L. gasseri*, and *L. paracasei*, which had bacterial counts ranging from 7.30 to 8 log CFU/mL after 24 h of fermentation. This finding is consistent with the present study, which also observed higher viability of *L. plantarum* in FCM (Table 1). In addition, the decrease in Lactobacillus strain counts during storage may be linked to FCM post-acidification (Figure 1), leading to a further drop in pH values [18,23]. 

Previous studies indicated that co-culturing *S. thermophilus* with Lactobacillus strains could improve its survival rate due to synergistic effects [15,26]. Santos et al. [28] noted that such interactions could enhance the viability of *S. thermophilus*, a trend confirmed by Wu et al. [29], who reported enhanced viability of *S. thermophilus* in fermented dairy products when co-cultured with Lactobacillus strains. Shori [30] also found that the interaction between Lactobacillus species and *S. thermophilus* promoted better survival of the latter during storage. The high preservation of viable cell counts in FCM over extended storage periods (Table 1) could enhance its probiotic potential. Compared with our previous study on yogurt made from cow milk using similar Lactobacillus starter cultures, *L. plantarum*-FCM maintained a higher overall VCC of *Lactobacillus spp* compared with cow milk yogurt [15].

### 3.3. Antioxidant Activity in FCM

**Figure 3:**
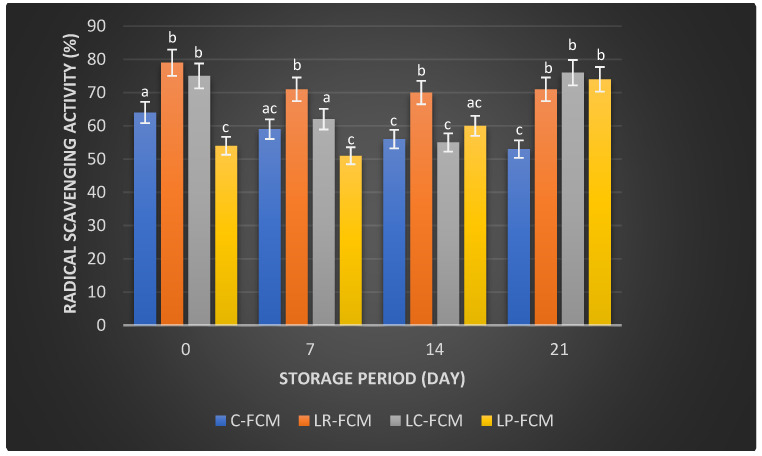
Radical scavenging activity (%) of fermented camel milk (FCM) with the addition of *L. rhamnosus* (Lr), *L. casei* (Lc), or *L. plantarum* (Lp) compared with control (C) FCM over 21 days of refrigerated storage at 4 °C. Data are presented as mean ± SEM. abc means with different superscript letters indicate the level of significance at *p* < 0.05 compared with control at the same storage period.

**Figure 4:**
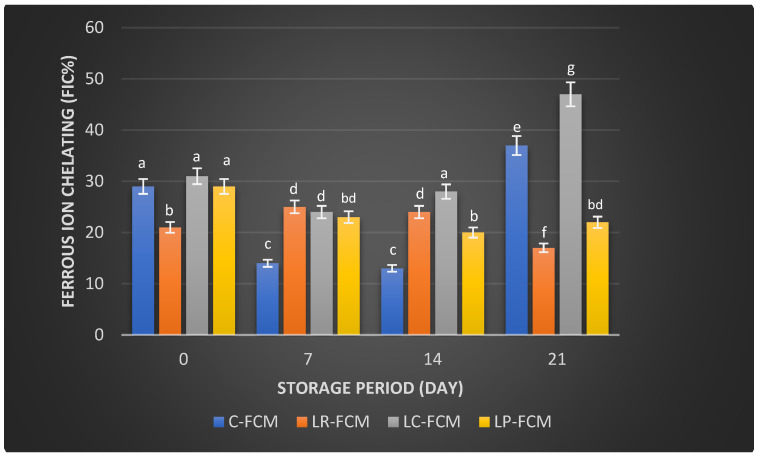
Ferrous ion-chelating (FIC; %) of fermented camel milk (FCM) with the addition of *L. rhamnosus* (Lr), *L. casei* (Lc), or *L. plantarum* (Lp) compared with control (C) FCM over 21 days of refrigerated storage at 4 °C. Data are presented as mean ± SEM. abcdefg means with different superscript letters indicate the level of significance at *p* < 0.05 compared with control at the same storage period.

**Figure 5:**
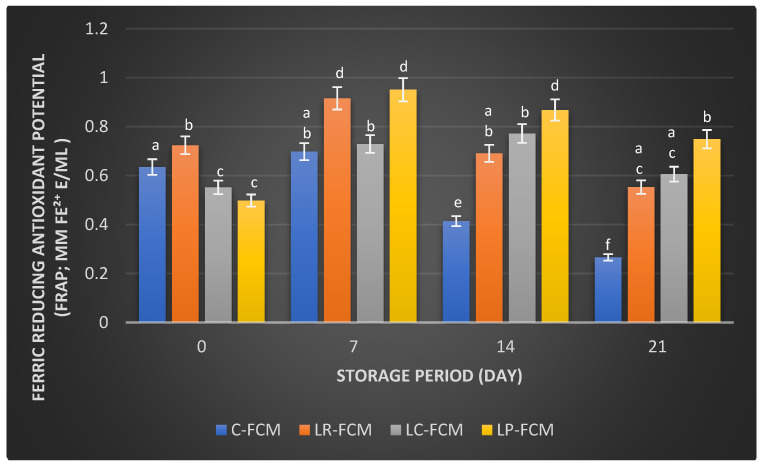
Ferric reducing antioxidant potential (FRAP; mM Fe^2+^ E/mL) of fermented camel milk (FCM) with the addition of *L. rhamnosus* (Lr), *L. casei* (Lc), or *L. plantarum* (Lp) compared with control (C) FCM over 21 days of refrigerated storage at 4 °C. Data are presented as mean ± SEM. abcdef means with different superscript letters indicate the level of significance at *p* < 0.05 compared with control at the same storage period.

Figure 3, Figure 4 and Figure 5 present the changes in the radical scavenging activity, Ferrous Ion Chelating (FIC), and ferric reducing antioxidant potential (FRAP), respectively, of FCM using various Lactobacillus starter cultures (*L. rhamnosus*, *L. casei*, and *L. plantarum*) compared with control FCM over 21 days of cold storage at 4 °C. On day 1, the control FCM exhibited a radical scavenging activity of 64 ± 0.004%, which progressively decreased to 53 ± 0.014% (*p* < 0.05) by day 21 (Figure 3). In contrast, FCM with *L. rhamnosus* started with the highest activity at 79 ± 0.051%, dropped (*p* < 0.05) to 70 ± 0.011% by day 14, and slightly increased (*p* > 0.05) to 71 ± 0.002% by day 21. The sample with *L. casei* showed an initial activity of 75 ± 0.001%, which decreased (*p* < 0.05) to 55 ± 0.001% by day 14 before rising (*p* < 0.05) to 76 ± 0.003% by day 21 (Figure 3). The FCM with *L. plantarum* started at 54 ± 0.002% and experienced a reduction (*p* > 0.05) to 51 ± 0.017% by day 7, but then it increased (*p* < 0.05) to 74 ± 0.003% by day 21 (Figure 3).

For Ferrous Ion Chelating (FIC), the control FCM exhibited an FIC activity of 29 ± 0.002% on day 1, which decreased (*p* < 0.05) by ~15% on days 7 and 14 of storage, and subsequently increased significantly (*p* < 0.05) to 37 ± 0.095% by day 21 (Figure 4). In contrast, the FCM with *L. rhamnosus* started with an FIC activity of 21 ± 0.001%, increased by ~3% during two weeks of storage, and further decreased (*p* < 0.05) to 17 ± 0.004% by day 21. The sample with *L. casei* showed an initial FIC activity of 31 ± 0.012%, which decreased (*p* < 0.05) to 24 ± 0.04% by day 7 and further increased (*p* < 0.05) by 4% and 23% on days 14 and 21, respectively (Figure 4). The FCM with *L. plantarum* started at 29% ± 0.02, decreased up to 20% ± 0.004 (*p* < 0.05) after 2 weeks, and slightly increased (*p* > 0.05) to 22% ± 0.001 by day 21.

For the FRAP assay, the control FCM exhibited a FRAP value of 0.635 ± 0.004 mM Fe^2^⁺ E/mL (day 1), which increased (*p* < 0.05) to 0.698 ± 0.003 mM Fe^2+^ E/mL by day 7 (Figure 5). However, this value was significantly decreased to 0.414 ± 0.040 and 0.266 ± 0.020 mM Fe^2^⁺ E/mL by days 14 and 21, respectively. In contrast, the FCM samples with *L. rhamnosus*, *L. casei*, and *L. plantarum* showed initial FRAP values of 0.724 ± 0.001, 0.552 ± 0.001, and 0.498 ± 0.001 mM Fe^2+^ E/mL, respectively (Figure 5). These results increased (*p* < 0.05) to 0.729–0.951 mM Fe E/mL, with the lowest value (*p* < 0.05) shown for *L. casei*-FCM on day 7 (Figure 5). A significant reduction in FRAP value occurred in *L. rhamnosus*-FCM (0.691 ± 0.007 mM Fe^2+^ E/mL), but there were no changes (*p* > 0.05) in L. *casei*- and *L. plantarum*-FCM on the 14th day of storage (Figure 5). All three *Lactobacillus* spp. FCMs further decreased to values ranging between 0.553 and 0.749 mM Fe^2^⁺ E/mL, with no significant differences between *L. rhamnosus*- and *L. casei*-FCM.

Camel milk naturally contains various antioxidants, including vitamins C and E, and certain peptides that can neutralize free radicals, reducing oxidative stress in the body [31]. Fermentation can enhance these antioxidant properties by increasing the bioavailability of these compounds and generating new antioxidant peptides through the activity of microbial enzymes [32]. Our observation found that some FCM with *L. rhamnosus*, *L. casei*, and *L. plantarum* maintained higher antioxidant levels than the control during storage (Figure 3, Figure 4 and Figure 5). These findings were aligned with previous findings that demonstrated the positive effects of Lactobacillus strains on the antioxidant properties of fermented dairy products [15,23,24,33]. The significant FIC activity of the FCM with *L. casei* by day 21 (Figure 4) underscored this starter culture’s potential to enhance fermented camel milk health benefits through improved Ferrous Ion Chelating capacity over time. Investigations have demonstrated that the antioxidants of camel milk can be further improved by fermentation processes involving specific probiotic strains, such as Lactobacillus [23,32]. Yogurt made from camel milk using Lactobacillus strains has been shown to have higher radical scavenging activity and FRAP compared with non-fermented camel milk [34]. Camel milk fermented with *L. acidophilus* DSM9126 demonstrated low DPPH antioxidant activity, ranging from 2% to 11% over 21 days of storage [33]. Similarly, camel milk yogurt fermented with a combination of 2% yogurt culture and 5% probiotic culture of *Bifidobacterium bifidum* showed a significant reduction in DPPH antioxidant activity, decreasing from 6% to 4% during two weeks of storage [35]. Conversely, Soleymanzadeh et al. [25] indicated that fermented camel milk with *L. plantarum* SM06 exhibited DPPH antioxidant activity of nearly 40% after 24 h of fermentation. Additionally, camel milk yogurt fermented with a yogurt culture composed of *S. thermophilus* and *L. delbrueckii ssp. bulgaricus* showed DPPH antioxidant activity ranging from 14.5% to 19% over two weeks [20]. However, the study demonstrated significantly higher DPPH antioxidant activity in FCM made with different Lactobacillus starter cultures, ranging from 51% to 79% over 21 days of storage, with *L. rhamnosus* being the most effective, followed by *L. casei* and *L. plantarum* as the least effective (Figure 3). A previous study demonstrated that the incorporation of *Lb. rhamnosus* B-442 and *Lb. rhamnosus* B-1445 with a commercial starter culture into fermented camel milk resulted in DPPH radical scavenging activity ranging from approximately 30% to 84% over a two-week storage period [36]. Fermentation not only increases the bioavailability of antioxidant compounds but also introduces new bioactive peptides with potent antioxidants [19]. Previous studies found that lactic acid bacteria, including *L. rhamnosus*, *L. casei*, and *L. plantarum*, possess the potential to function as reducing agents, hydrogen donors, and quenchers of singlet oxygen [15,16]. This capability may stem from their fermentation byproducts, such as bioactive peptides, amino acids, vitamins, minerals, and volatile acids, which contribute to their antioxidant properties [6]. In addition, the antioxidant activity in fermented milk is likely influenced more by the specific bacterial strains used and the unique action of their proteolytic enzymes, rather than by ongoing protein breakdown or bacterial proliferation [37]. The production of antioxidant peptides and other bioactive compounds is closely linked to the metabolic activities of these lactic acid bacteria, which can vary significantly between different species and even among strains of the same species. Further research is required to explore the presence and role of bioactive peptides in FCM with antioxidant capabilities. Surprisingly, compared with our previous study on yogurt made from cow milk using similar Lactobacillus starter cultures, cow milk yogurt demonstrated higher radical scavenging activity and relatively stable FIC values than FCM [15]. However, FCM showed higher FRAP values than cow milk yogurt. Although commercially available camel milk was used, factors such as the severity of pasteurization, stage of lactation, feeding management of animals, milk quality, and storage conditions can all impact its antioxidant content [38].

### 3.4. Sensory Evaluation of FCM

**Figure 6:**
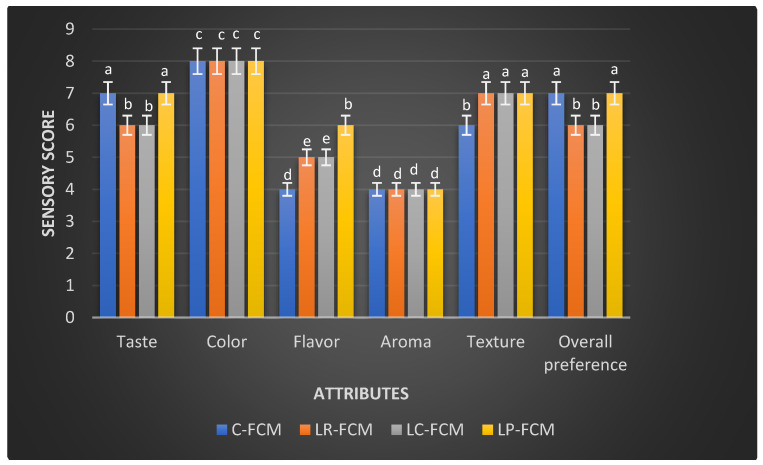
Sensory evaluation of fresh fermented camel milk (FCM) with the addition of *L. rhamnosus* (Lr), *L. casei* (Lc), or *L. plantarum* (Lp) compared with control (C) FCM over 21 days of refrigerated storage at 4 °C. Data are presented as mean ± SEM. abcde means with different superscript letters indicated the level of significance at *p* < 0.05 compared with control at the same storage period.

The FCM was subjected to a sensory evaluation on the initial day of cold storage at 4 °C (Figure 6). Both the control and the three treated FCM scored similarly for color (8: acceptable) and aroma (4: moderately unacceptable; Figure 6). However, the flavor scores differed, with *L. rhamnosus* and *L. casei* scoring slightly unacceptable, whereas *L. plantarum* achieved the highest flavor score (6: slightly acceptable; *p* < 0.05) compared with the control (4: moderately unacceptable). Additionally, the presence of the three Lactobacillus strains significantly improved the FCM texture (7: moderately acceptable) compared with the control (6: slightly acceptable (*p* < 0.05); Figure 6). The FCM with *L. plantarum* also showed comparable scores to the control for taste and overall preference (7: moderately acceptable).

The sensory perception discipline utilizes the human senses to provide reliable data to inform product development, quality control, and market research, ensuring that food products meet consumer expectations and standards. In this study, the results are consistent with previous studies that demonstrated the influence of different Lactobacillus strains on the sensory properties of fermented dairy products [15,16]. According to Li et al. [22] and Sun et al. [39], Lactobacillus-fermented milk products often exhibit improved texture and flavor profiles, which was aligned with higher texture scores in the presence of the three *Lactobacillus* spp. in our study (Figure 6). The increased acidity can affect the texture and taste of the fermented milk [40]. FCM-*L. plantarum* showed the highest pH values on day 1 (Figure 1), which could lead to a less tart taste. Furthermore, Li et al. [22] observed that the incorporation of *L. plantarum* could enhance the overall sensory acceptability of dairy products such as yogurt. This finding aligns with our results, where FCM containing *L. plantarum* received the highest overall preference score among the tested samples (Figure 6). Similarly, Soleymanzadeh et al. [25] reported that *L. plantarum* SM06 improved the texture score of fermented milk after 24 h of fermentation. These findings highlight the potential of using specific Lactobacillus starter cultures to enhance the sensory attributes of fermented camel milk, thereby improving its acceptability and marketability. However, aroma weaknesses in fermented camel milk products, particularly those produced by Lactobacillus spp., can be linked to proteolytic activity, a critical process during fermentation [41]. Proteolytic activity involves the breakdown of milk proteins, such as caseins, by enzymes produced by Lactobacillus strains, leading to the formation of bioactive peptides [41]. While these peptides contribute to the health benefits of fermented milk, they can also lead to the development of undesirable off-flavors and aromas during fermentation [41]. To address aroma and flavor challenges, manufacturers could incorporate natural additives such as herbs, spices, or fruit extracts, which may enhance aroma and help modulate the flavor profile [7,12,18,35,40].

## 4. Conclusions

The findings of this study highlight the beneficial effects of incorporating *L. rhamnosus*, *L. casei*, and *L. plantarum* into fermented camel milk. These *Lactobacillus* strains significantly enhanced the antioxidant properties and maintained higher viable cell counts throughout the storage period, thereby increasing the fermented camel milk’s health benefits. Sensory evaluation revealed that FCM containing these Lactobacillus strains was well accepted, especially in terms of flavor and texture. Specifically, *L. plantarum* significantly enhanced the flavor, texture, and overall preference, suggesting its potential as a functional food product. Further research is required to explore the proteolytic activity of these strains in camel milk throughout fermentation and storage, as well as to assess the potential presence of bioactive peptides with health benefits.

## Figures and Tables

**Table 1:** Viable cell counts (VCC) of *S. thermophilus* and *Lactobacillus* spp. in fermented camel milk (FCM) with the addition of *L. rhamnosus* (Lr), *L. casei* (Lc), or *L. plantarum* (Lp) compared with control (C) FCM during 21 days of refrigerated storage at 4 °C.

VCC (log CFU/mL).								
Sample	*S. thermophilus*				*Lactobacillus* spp.			
	0 Day	7-Day	14-Day	21-Day	0-Day	7-Day	14-Day	21-Day
**C-FCM**	8.15 ± 6.42 ^a^	8.15 ± 4.72 ^a^	7.95 ± 6.08 ^ab^	6.00 ±0.57 ^d^	6.00 ± 0.57 ^a^	6.23 ± 4.16 ^b^	6.20 ± 3.60 ^b^	6.04 ± 1.52 ^a^
**Lr-FCM**	8.63 ±1.63 ^a^	8.82 ± 1.77 ^a^	8.79 ±3.96 ^ac^	8.54 ± 3.32 ^ac^	6.35 ± 0.21 ^b^	6.67 ± 4.17 ^cd^	6.41 ± 0.14 ^be^	6.13 ± 0.35 ^b^
**Lc-FCM**	8.64 ±1.91 ^a^	8.68 ±4.74 ^a^	8.48 ±2.05 ^ab^	8.15± 0.28 ^ab^	6.87 ± 3.25 ^c^	6.41 ±1.34 ^b^	6.33 ±1.34 ^b^	6.28 ± 0.14 ^b^
**Lp-FCM**	8.43 ±1.13 ^a^	8.73 ±4.81 ^a^	8.27 ±0.78 ^ab^	8.29 ±0.35 ^ab^	6.94 ± 0.49 ^c^	6.70 ± 1.20 ^cd^	6.63 ± 2.47 ^cd^	6.50 ± 3.04 ^cd^

Data are presented as mean ± SEM. Different letters in the same column means significantly different (*p* < 0.05).

## Data Availability

The original contributions presented in the study are included in the article, further inquiries can be directed to the author.
